# Succeeding in Academic Medicine: A Roadmap for Diverse Medical Students and Residents

**DOI:** 10.5195/jmla.2021.1245

**Published:** 2021-07-01

**Authors:** Len Levin

**Affiliations:** 1 leonard_levin@hms.harvard.edu, Francis A. Countway Library of Medicine, Harvard Medical School, Boston, MA

*Succeeding in Academic Medicine* would be an excellent addition to works assisting those early in their careers in medicine having a desire to move into a teaching role. Where it fails is reflected in the book's subtitle, *A Roadmap for Diverse Medical Students and Residents.*

The book is edited by John P. Sánchez, the executive director of Latino Medical Students Association and president of Building the Next Generation of Academic Physicians, Inc. He is joined by twenty-two contributors, many of whom are also reflective of diverse communities including Latinx, African American, and LGBTQ+ (lesbian, gay, bisexual, transgender, and questioning/queer plus). Each chapter includes a personal reflection by its contributors discussing their backgrounds and how they found successful careers in academic medicine. Chapters within the book include research and scholarship, significance of mentorship, finding an academic residency program, finding an academic position after residency, and financing a career in academic medicine. Many chapters contain case studies meant to engage the reader in ways the case subject used or could have used specific research or leadership methods to reach their project or career goals. These case studies focus on subjects who are early in their medical career. Although it is not specifically cited in many of the cases, we are left to imagine that these subjects are from various diverse communities.

While the book does a good job of including data showing that academic medicine is today still mainly male and white and spelling out problems faced by those from diverse communities looking to a career in academic medicine, it does little to provide answers to members of these communities. For example, while we are led to believe the subjects in the case studies are from diverse communities, they could reflect any population within a medical education environment.

Throughout the book, two concepts are often mentioned: minority tax and impostor syndrome. Minority tax is defined as an additional burden placed upon minority faculty members to participate in committees to ensure reflective diversity of the group. Impostor syndrome is a psychological condition for which an individual, in this case from a diverse background, believes they do not belong even though they hold identical credentials and educational attainment to others in the group. However, the advice highlighted in the chapters and case studies suggest avenues such as finding a good mentor or using one of a handful of validated service project models for community outreach—advice that would be practical for anyone desiring a career in academic medicine. As for the minority tax and impostor syndrome, while mentioned several times, nowhere in the book are there any suggestions given specifically for persons from diverse communities on how they might overcome these psychological issues.

In chapter 9, a contributor discusses how living in a poor West Coast community gave him a clearer sense of the struggles that many Americans and his future patients face. Another contributor in chapter 1 discusses finances and an academic career and says,

Initially in your career, when you had no children, your earning potential may not have been as important. But now that you have children, it has required you to move to a community that not only has a better school district with excellent educational opportunities but has necessitated that you live in a neighborhood where the cost of living in much higher … (p. 9).

This is a classic example of pulling up the ladder after you have ascended it and is tantamount to an insult to members of diverse communities who often have little choice as to the communities where they are able to live.

The book is written in the second person as opposed to the third person that is most common is scholarly literature. Some might find this method colloquial or even “folksy,” but it often came across to me as pedantic. For any reader looking to advance their career in academic medicine, this work succinctly lays out the steps one must take and pitfalls one must avoid to find themselves a suitable and rewarding career in medicine. For members of diverse communities who, as it is often noted, work above and beyond to advance their careers, a separate work, possibly with expanded personal narratives from the contributors highlighting their struggles and successes, could be more helpful.

